# Simultaneous Quantification of 3′- and 6′-Sialyllactose in Rat Plasma Using Liquid Chromatography-Tandem Mass Spectrometry and Its Application to a Pharmacokinetic Study

**DOI:** 10.3390/molecules26041177

**Published:** 2021-02-22

**Authors:** Seok-In Jang, Han Young Eom, Jeong Ho Hwang, Lila Kim, Jong-Hwa Lee

**Affiliations:** 1 Bioanalytical and Pharmacokinetic Study Group, Korea Institute of Toxicology, Daejeon 34114, Korea; sijang@kitox.re.kr (S.-I.J.); hanyoung.eom@kitox.re.kr (H.Y.E.); 2 Animal Model Research Group, Jeonbuk Department of Inhalation Research, Korea Institute of Toxicology, Jeongeup, Jeollabuk-do, Daejeon 53212, Korea; jeongho.hwang@kitox.re.kr; 3 GeneChem Inc., A-501, 187 Techno 2-ro, Daejeon 34025, Korea; lilakim@genechem.co.kr

**Keywords:** sialyllactose, method validation, HILIC column, mass spectrometry, surrogate matrix

## Abstract

Sialyllactose (SL), an acidic oligosaccharide, has immune-protective effects against pathogens and helps with the development of the immune system and intestinal microorganisms. To elucidate the pharmacokinetic characterization after oral administration to rats, the simultaneous quantification method for 3′-SL and 6′-SL in rat plasma was validated, using liquid chromatography-tandem mass spectrometry (LC-MS/MS) in an electrospray ionization (ESI) mode. Several types of columns [C18, amide, and hydrophilic interaction liquid chromatography (HILIC) phase] were used to separate the peaks of 3′-SL and 6′-SL, which improved chromatographic selectivity. Ultimately, the HILIC phase column had a good peak shape and quick resolution, with a mobile phase comprising ammonium acetate buffer and acetonitrile obtained by gradient elution. In addition, the simultaneous quantification of 3′-SL and 6′-SL in rat plasma samples were adequately applied to pharmacokinetic study.

## 1. Introduction

Sialyllactose (SL), a kind of sialyloligosaccharide, is an oligosaccharide with sialic acid and a lactose group [1]. The most abundant sialyloligosaccharides in human milk are 3′-sialyllactose (3′-SL), 6′-sialyllactose (6′-SL), sialyllacto-*N*-tetraose (SLNT), and disialyllactose-*N*-tetraose (DSLNT) [2,3]. SL is being developed as a chemically synthesized medication for osteoarthritis, as it promotes cartilage formation and effectively inhibits cartilage destruction.

Human milk oligosaccharides (HMOs) have low sensitivity to UV detection and are not well retained in reversed-phase analytical columns, such as the C18 column. Most of the conventional methods measured HMO levels in milk, and only Santos-Fandila’s method analyzed sialyllactose levels in plasma or serum. HMOs in serum were extracted into chloroform/methanol (2:1, *v*/*v*) and were precipitated by ethanol, overnight, at 4 °C. The sample preparation steps were simple. We used only methanol, not highly toxic chloroform. Conventional mass spectrometry methods are limited in terms of quantitative analysis. In addition, the time for sample preparation was reduced, as compared to that of the overnight method. 

Thus, the measurement of 3′- and 6′-SL in rat plasma was developed, using LC–MS/MS. The method requires highly sensitive and relatively little analytical run-time. We found endogenous sialyllactose in rat plasma, and a calibration curve spiked standard solution in a blank matrix cannot be used, since 3′- and 6′-SL are endogenous compounds. To overcome this huddle, background subtraction, the standard addition, and a surrogate matrix and surrogate analyte were used. The surrogate matrix method had benefits, compared to other methods, since it employs a labeled internal standard (IS) and enables the evaluation of parallelism [4,5]. This study was performed to verify the bioanalytical method, including the guidelines of the United States Food and Drug Administration (FDA) [6].

## 2. Results and Discussion

### 2.1. Separation of 3′-SL and 6′-SL and Extraction

**Figure 1:**
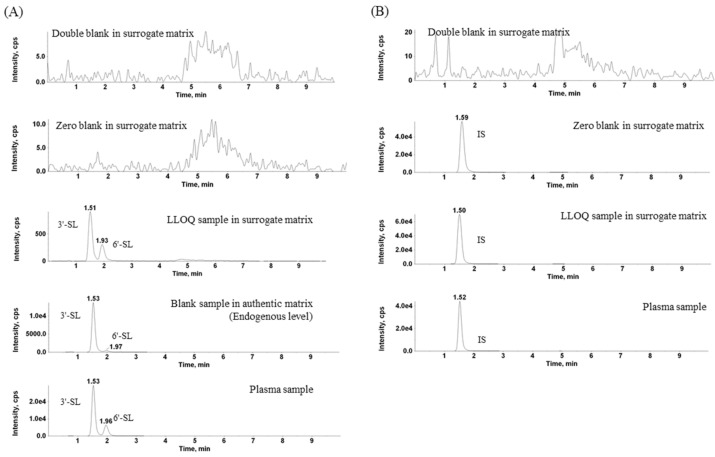
Extracted multiple-reaction monitoring (MRM) chromatograms at (**A**) *m/z* 632.4→290.0 for 3′-SL (sialyllactose) and 6′-SL; double blank, zero blank, and lower limit of quantification (LLOQ) in the surrogate matrix, blank sample in authentic matrix, and rat plasma sample and (**B**) *m*/*z* 636.2→293.0 for internal standard; double blank, zero blank, and LLOQ sample in the surrogate matrix, and rat plasma sample.

The separation of 3′-SL and 6′-SL in rat plasma was optimized with reference to several articles [5,6,7,8,9] (Figure 1A). The HILIC column (2.1 mm × 50 mm, 3 µm; waters) was finally selected, because it had the best selectivity. The gradient profile was 0.0–1.0 min, 83% B; 1.0–1.1 min, 83–50% B; 1.1–3.0 min, 50% B; 3.0–3.1 min, 50–83% B; and 3.1–10 min, 83% B, and the flow rate was set to 0.3 mL/min. Considering the sensitivity, resolution, asymmetry, and the buffer capacity of ammonium acetate ranging from pH 3.8 to 5.8, the mobile phase was set to 10 mM ammonium acetate buffer (pH 4.5) and the acetonitrile (Figure 1B).

Methanol was selected for protein precipitation in rat plasma samples. To ensure compatibility to the HILIC column, the methanol extract was totally evaporated with a vacuum concentrator and reconstituted with the solution comprising 10 mM ammonium acetate (pH 4.5) and acetonitrile (40:60, *v*/*v*), as this afforded well-shaped peaks and the best sensitivity.

### 2.2. Method Validation

#### 2.2.1. Selectivity

**Figure 2:**
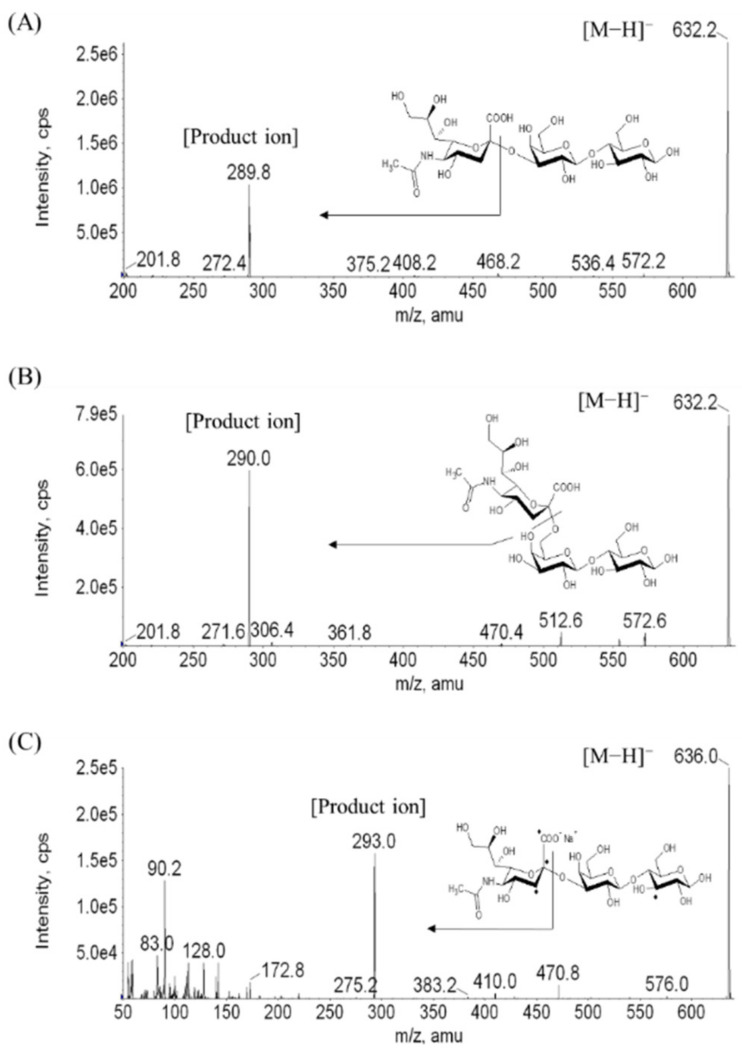
Molecular structure and product-ion scan spectra of (**A**) 3′-SL, (**B**) 6′-SL, and (**C**) internal standard (IS).

Chemical structure and product ion scan spectra of 3′-SL, 6′-SL, and internal standard are shown in Figure 2. Comparing the peak area ratios of quantification and qualification ion transitions between the medium quality control (MQC) samples in plasma and the surrogate matrix, the selectivity was monitored. The *m/z* values for quantification and qualification was 632.4→290.0 and 632.4→572.2, respectively (Table 1). The MQC sample in the surrogate matrix was made from spiking the 3′-SL and 6′-SL standards to surrogate matrix at concentration of 600 ng/mL. No marked difference was observed in the peak area ratios between the quantification and qualification ion transitions. In addition, interference of 3′-SL and 6′-SL at the retention time was not found in the surrogate matrix (water). 

#### 2.2.2. Linearity, Accuracy and Precision

The linearity in surrogate matrix was estimated from calibration curve as evidenced by a correlation coefficient of at least 0.999 in both 3′-SL and 6′-SL by a 1/x^2^ weighting factor from 20 to 10,000 ng/mL. The accuracy of calibration in the standard samples for 3′-SL and 6′-SL ranged from −1.7% to 2.2% and from −2.5% to 2.3%, respectively, which met to the acceptance criteria of being within ±15% (±20% for LLOQ) of the nominal concentration. In intra-day and inter-say accuracy (RE, %) and precision (CV, %), the method was satisfied the acceptance criteria of being within ±15% (±20% for LLOQ) (Table 2).

#### 2.2.3. Matrix Effect and Recovery

In this study, the matrix effect and recovery were estimated as the IS-normalized method. MQC and HQC (600 and 7500 ng/mL) were prepared with six different blank samples spiking the standard solution. Mean values of the matrix effect for six individual samples were 113.9% and 103.1% in MQC and HQC for 3′-SL, and 101.9% and 108.4% in MQC and HQC for 6′-SL, respectively (Table 3). In addition, precision (CV, %) of matrix effect satisfied the acceptance criteria (within ±15%).

Recovery also was evaluated at two quality control (QC) samples (600 and 7500 ng/mL). Mean values of recovery ranged from 88.6% to 94.7% for 3′-SL and from 89.0% to 91.4% for 6′-SL, respectively. In addition, precision (CV, %) of recovery satisfied the acceptance criteria (within ±15%).

#### 2.2.4. Parallelism

The equations of calibration curve plotted with the standard addition method were y = 0.000604x + 0.292 for 3′-SL and y = 0.000257x + 0.0117 for 6′-SL. The endogenous concentrations in the authentic matrix were 483 and 45.5 ng/mL as the x-intercept for 3′-SL and 6′-SL, respectively. Using the calibration curve in the surrogate matrix, we analyzed QC samples at concentration of 60, 600, and 7500 ng/mL in the authentic matrix, in six replicates. Accuracy (RE, %) ranged from −7.8% to −5.4% for 3′-SL and from −7.4% to 2.8% for 6′-SL, and precision (CV, %) ranged from 1.4% to 2.2% for 3′-SL and from 2.7% to 7.6% for 6′-SL (Table 4), indicating that the surrogate matrix can be applied to analyze rat plasma.

#### 2.2.5. Carryover and Dilution Integrity

The blank sample following the upper LOQ (ULOQ) sample at a concentration of 10,000 ng/mL in the surrogate matrix was analyzed, to evaluate the carryover. The peaks of 3′-SL, 6′-SL, and IS in the blank sample were not detected at the retention times. A dilution quality control was prepared, with six replicates, at 50,000 ng/mL, in the blank plasma. The accuracy and precision of 10-fold diluted samples were evaluated. The accuracy (RE) of diluted samples was −6.0% and −0.3% for 3′-SL and 6′-SL, respectively, and precision (CV) was 0.7% and 0.8% for 3′-SL and 6′-SL, respectively. These results satisfied the acceptance criteria (within ±15%) and showed that plasma samples over the ULOQ can be analyzed.

#### 2.2.6. Stability

The stability was evaluated, using LQC (3′-SL, 481 ng/mL; 6′-SL, 37.5 ng/mL) and HQC (7500 ng/mL). The value of accuracy (RE, %) and precision (CV, %) for the benchtop stability of QC samples, for 4 h, at room temperature, was −8.1% to −3.7% and 1.6% to 4.3% for 3′-SL, respectively, and −1.3% to −0.1% and 4.6% to 7.1% for 6′-SL, respectively. The accuracy (RE) and precision (CV) for the post-preparative stability of QC samples for 26 h, at 10 °C, on an HPLC autosampler tray, was −5.5% to 0.1% and 1.6% to 3.3% for 3′-SL, respectively, and −7.4% to −3.9% and 0.6% to 2.8% for 6′-SL, respectively. For freeze-and-thaw stability, QC samples were frozen and thawed from −80 °C to room temperature, in four cycles. The RE and CV of QC samples ranged from −8.0% to −3.5% and 0.5% and 3.2% for 3′-SL, respectively, and −4.3% to 3.9% and 2.2% to 11.5% for 6′-SL, respectively. The long-term QC samples at −80 °C were stable for 37 days, with accuracy of −10.0% to −3.4% and precision of 2.3% to 5.1% for 3′-SL, and with accuracy of −6.4% to −5.4% and precision of 1.3% to 3.7% for 6′-SL (Table 5).

Therefore, all stability of 3′-SL and 6′-SL in the biological matrix were verified under various storage conditions. 

### 2.3. Application to a Pharmacokinetic Study in Rats

**Figure 3:**
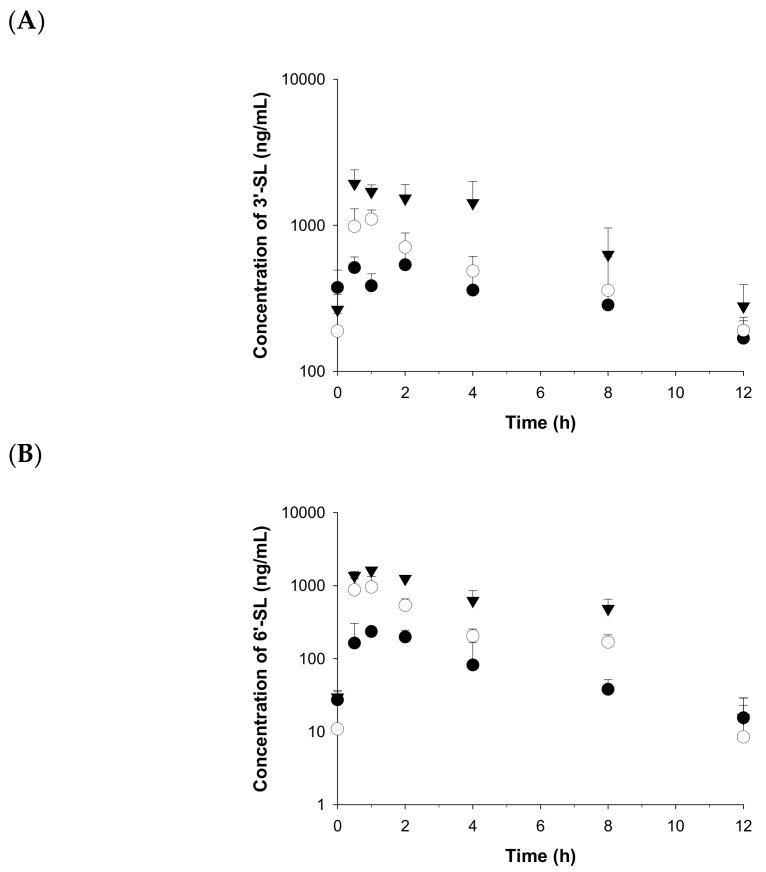
Temporal profiles of plasma concentration of (**A**) 3′-SL and (**B**) 6′-SL in rats, after intravenous administration at doses of 100 (●), 500 (○), and 2000 (▲) mg/kg. Data are expressed as the mean ± SD of triplicate runs.

The mean plasma concentration–time curves in male rats following oral administration of 3′-SL and 6′-SL at doses of 100, 500, and 2000 mg/kg are plotted in Figure 3. In pharmacokinetic parameters, both 3′-SL and 6′-SL were eliminated with half-lives (t_1/2_) ranging from 3.4 to 6.5 h for 3′-SL and from 1.8 to 2.8 h for 6′-SL (Table 6). The 6′-SL more rapidly decreased than 3′-SL in the terminal phase. Finally, the AUC_last_ values were 4000, 5700, and 12,000 ng·h/mL for 3′-SL and 991, 3270, and 7620 ng·h/mL for 6′-SL at doses of 100, 500, and 2000 mg/kg, respectively, and 3′-SL was present at 1.6- to 4.0-fold higher quantities than 6′-SL at the same doses. The systemic clearance (CL) values ranged from 18,000 to 150,000 mL/h/kg for 3′-SL and from 95,000 to 261,000 mL/h/kg for 6′-SL and changed significantly as increasing dose, indicating that 3′-SL and 6′-SL were eliminated with non-linear characterization at doses ranging from 100 to 2000 mg/kg, with saturable kinetics. In addition, the V_d_ of 3′-SL and 6′-SL was extremely high, 168,000–737,000 and 383,000–687,000 mL/kg, respectively, supporting non-linear kinetics for 100–2000 mg/kg doses and explaining that the tissue distribution from blood can be significantly high, considering the amount of body fluid physiologically. Therefore, for the further study, to clarify the tissue distribution and plasma pharmacokinetics, intravenous administration in rats is needed at the low-dose level. 

## 3. Materials and Methods

### 3.1. Chemicals and Reagents

The 3′-SL (98.55%) and 6′-SL (98.75%) were provided from GeneChem (Daejeon, Korea). An internal standard, [1,2,3-^13^C_3_]3′-sialyl[3-^13^C^glc^]lactose sodium salt (97.00%), was purchased from Omicron Biomedicals (South Bend, IN, USA). Methanol and acetonitrile (HPLC grade) were purchased from Burdick & Jackson (Muskegon, MI, USA). Acetic acid (HPLC grade) was purchased from Sigma-Aldrich (St. Louis, MO, USA). Ammonium acetate (HPLC grade) was purchased from Fluka (Muskegon, MI, USA). Purified water was obtained with the Elix & Milli-Q Biocel system (Milford, MA, USA). 

### 3.2. HPLC–MS

Agilent HP1200 HPLC system (Santa Clara, CA, USA), consisting of a degasser, binary pump, autosampler, column oven, and a Waters Atlantis HILIC column (50 mm × 2.1 mm, 3 µm; Milford, MA, USA) was used for chromatographic separation. The mobile phase A and B consisted of (A) 10 mM ammonium acetate in water (pH 4.5) and (B) acetonitrile, following the gradient profile 0.0–1.0 min, B 83%; 1.0–1.1 min, B 83%–50%; 1.1–3.0 min, B 50%; 3.0–3.1 min, B 50%–83%; and 3.1–10 min, B 83%. The flow rate of the mobile phase was at 0.3 mg/min, and the column oven was maintained at 40 °C.

Mass spectrometer (MS) was performed with an AB Sciex API 4000 MS/MS system (Framingham, MA, USA) equipped with an electro-spray ionization source operating in the negative ion mode. The MRM mode was used to monitor the analyte. The curtain gas, collision gas, nebulizing gas (GS1), and drying gas (GS2) were set to 15, 6, 50, and 50 psi, respectively. The ion spray voltage and ion source temperature were −4500 V and 500 °C, respectively. The de-clustering potential, entrance potential, and collision energy were optimized at −94 V, −10 V, and −37 eV, respectively. The ion transitions of SL were monitored at *m/z* 632.4→290.0 for quantification and *m*/*z* 632.4→572.2 for qualification ion transition. The quantification ion transition of the IS was *m*/*z* 636.2→293.0 (Figure 1). The data were acquired and processed with Analyst (Version 1.4.2; AB Sciex) software.

### 3.3. Preparation of Standard Stock Solutions and Quality Control Samples

Each standard stock solution of 3′-SL and 6′-SL was prepared separately by dissolving in purified water (0.5 mg/mL). To prepare working solutions of 3′-SL and 6′-SL, standard stock solution was diluted serially to water. A stable isotope substituted SL of [1,2,3-^13^C_3_]3′-sialyl[3-^13^C^glc^]lactose sodium salt was used as an internal standard, and diluted to water at concentration of 0.1 mg/mL. In this study, purified water was used as a surrogate matrix instead of rat plasma, to prepare the calibration curve and LQC sample. To prepare quality control (QC) samples, a similar method was used at 60 (low QC in purified water), 600 (medium QC in the rat plasma), and 7500 nL (high QC in the rat plasma).

### 3.4. Sample Preparation

A rat plasma protein was precipitated, using methanol to extract SL. A 20 µL aliquot of rat plasma sample was transferred to a microcentrifuge tube. A 20 µL aliquot of IS solution at a concentration of 2000 ng/mL and 500 µL methanol were added to the tube, followed by vortexing for 10 min. Then, the mixture was centrifuged (16,363× *g* at 10 °C for 10 min), and the supernatant was transferred to speed-vacuuming (miVac Quattro concentrator, Genevac, Ipswich, UK), for evaporation at 45 °C, for 45 min. The residue was reconstituted with 100 µL of mobile phase A and B (4:6, *v*/*v*), and a 2 µL aliquot was injected onto the LC–MS/MS.

### 3.5. Method Validation

The LC–MS/MS procedure for quantifying 3′-SL and 6′-SL was validated in terms of selectivity, linearity, accuracy, precision, recovery, matrix effect, parallelism, carryover, dilution integrity, and stability, according to US Food and Drug Administration guidance [10].

#### 3.5.1. Selectivity

SL is an endogenous compound that is contained within a blank matrix. For assessment of the selectivity, one qualification ion transition was selected, and the peak area ratios of SL between quantification and qualification ion transitions were calculated. The peak area ratios between the MQC sample in the surrogate matrix and the real plasma samples were compared and evaluated for equality [11].

#### 3.5.2. Linearity, Accuracy, and Precision

Calibration curves in the concentration range from 20 to 10,000 ng/mL were made with the surrogate matrix. Accuracy and precision for intra-day and inter-day were evaluated at LLOQ and QC samples at concentrations of 20, 60, 600, and 7500 ng/mL. Six replicates at each level were analyzed on intra-day, to evaluate accuracy and precision. To determine inter-day accuracy and precision, the procedure was repeated over 3 days. The blank rat plasma samples were analyzed, to quantify the endogenous compound of each batch, and the results were used to revise the concentrations of QC and LLOQ samples. Precision is expressed by coefficient of variation (CV, %). Accuracy is expressed as relative error (RE, %).

RE (%) = (Determined conc. of QC − Endogenous conc.)/Nominal conc. × 100(1)

#### 3.5.3. Matrix Effect and Recovery

The matrix effect was assessed by normalizing with IS value, which is calculated the ratio of the peak area of the analyte and that of the IS. Comparing the value between the standard solution and six individual blank rat plasma samples spiked with a known analyte at the concentrations of MQC and HQC (600 and 7500 ng/mL), following sample extraction, we evaluated the IS-normalized value. The peak area in QC samples was revised by subtracting the peak area of endogenous SL in the rat plasma.

Recovery was determined by the variation in peak area at two concentrations (600 and 7500 ng/mL), with six replicate analyses at each concentration. We compared a blank sample spiked with a known concentration of analyte to the post-extraction samples with the sample-spiked analyte prior to sample extraction. The value was revised by subtracting the peak area of endogenous SL in the authentic matrix. The matrix effect and recovery were evaluated with precision, using six different QC samples for MQC and HQC.

#### 3.5.4. Parallelism

Parallelism to require for quantification of endogenous compounds was evaluated, using the accuracy (RE, %) and precision (CV, %) of QC samples in the authentic matrix by the calibration curve in the surrogate matrix [10,12,13,14,15].

#### 3.5.5. Carryover and Dilution Integrity

For carryover test, the blank sample in the surrogate matrix following the ULOQ sample at a concentration of 10,000 ng/mL in the surrogate matrix was analyzed, and the peak area of SL in the blank sample should be less than 20% of that of the LLOQ sample.

For the dilution test, the six replicates of the QC sample above the ULOQ were prepared at a concentration of 50,000 ng/mL, in the blank plasma, and diluted 10-fold with surrogate matrix, to 5000 ng/mL.

#### 3.5.6. Stability

The stability tests were performed in various circumstances, by measuring LQC (3′-SL, 481 ng/mL; 6′-SL, 37.5 ng/mL) and HQC (7500 ng/mL) samples with three replicates. The nominal LQC concentration set to the background concentration of SL in the blank authentic plasma samples. Adding a standard solution to the authentic matrix, we prepared the HQC samples, and the results were calculated by subtracting the endogenous concentration in the blank matrix. Benchtop stability, i.e., short-term stability, was assessed by allowing QC samples at room temperature (24 °C) for 4 h, and post-preparation stability stored on an autosampler tray was assessed at 10 °C for 26 h. The freeze–thaw stability was assessed over four cycles of thawing from −80 °C at room temperature, and long-term stability of stored QC samples at −80 °C was assessed for 37 days.

### 3.6. Application to a Pharmacokinetic Study in Rats

Male Sprague-Dawley rats were purchased from Orient Bio (Gyeonggi-do, Korea). The protocols were reviewed by the Institutional Animal Care and Use Committee (IACUC) and fully accredited by the Association for Assessment and Accreditation of Laboratory Animal Care International (AAALAC International) in the Korea Institute of Toxicology. All procedures were in compliance with the Animal Welfare Act and Guide for the Care and Use of Laboratory Animals (by ILAR publications).

A total of 27 seven-week-old male rats, weighing 243–277 g and divided into groups of 3 animals/time point/sex/group, were administered with 100, 500, and 2000 mg/kg oral doses of 3′-SL and 6′-SL. Dose volume was 10 mL/kg with distilled water. Blood samples (approximately 0.5 mL) were taken from the rat tail vein into K2-EDTA tubes at pre-dose (0) and 0.5, 1, 2, 4, 8, and 12 h post-dose and then gently mixed and placed on crushed wet ice/Kryorack until centrifugation. Plasma samples were obtained by centrifugation of the blood at 20,740× *g*, at 4 °C, for 3 min, and stored at −80 °C, prior to being analyzed.

The plasma concentration–time data were analyzed by the non-compartmental method, using Phoenix WinNonlin (Version 8.1; Certara, Princeton, NJ, USA, 2019). The maximum concentration (C_max_) and the time point (T_max_) at C_max_ were read directly from the time–concentration profile. The area under the concentration–time curve from time zero to the last quantifiable time point (AUC_last_) and the area under the concentration–time curve from time zero to infinity (AUC_inf_) were calculated, using the linear trapezoidal method and standard area extrapolation method [16]. Standard moment analysis was applied to calculate terminal half-life (t_1/2_), systemic clearance (CL), and volume of distribution (V_d_).

## 4. Conclusions

An LC–MS/MS procedure for quantifying 3′-SL and 6′-SL in rat plasma was a very fast and sensitive. The method was validated in terms of linearity, selectivity, accuracy, precision, recovery, matrix effect, carryover, dilution integrity, parallelism, and stability. The sample preparation steps were much simpler; we used only methanol, not highly toxic chloroform, and the sample preparation time also was reduced, compared to the overnight method. The sensitivity estimated as the LLOQ value was improved to 20 ng/mL from 60 ng/mL [5]. In addition, this quantification method for 3′-SL and 6′-SL used the surrogate matrix method, and it was successfully applied to a pharmacokinetic study in rats after administration of 3′-SL and 6′-SL at doses of 100, 500, and 2000 mg/kg. Therefore, the assay promises to be useful in the further preclinical study for development with 3′-SL and 6′-SL.

## Figures and Tables

**Table 1:** Selectivity for 3′-SL and 6′-SL.

	3′-SL	6′-SL
	PA_Quant ^a^/PA_Qual ^b^	PA_Quant/PA_Qual
MQC	108.6	1.745
Rat plasma 1	106.2	1.721
Rat plasma 2	106.5	1.747
Rat plasma 3	105.4	1.737
Rat plasma 4	111.1	1.683
Rat plasma 5	120.4	1.728
Rat plasma 6	101.6	1.742
Mean	108.5	1.729
CV(%) ^c^	5.5	1.3

^a^ PA_Quant = the peak area at the quantification ion transition (*m/z* 632.4→290.0). ^b^ PA_Qual = the peak area at the qualification ion transition (*m/z* 632.4→572.2). ^c^ CV (%) = standard deviation/mean × 100.

**Table 2:** Accuracy and precision for intra-day and inter-day.

	Nominal Concentration (ng/mL)							
	3′-SL				6′-SL			
	LLOQ	LQC	MQC	HQC	LLOQ	LQC	MQC	HQC
	20	60	600	7500	20	60	600	7500
*(A) Intra-day (n = 6)*								
Mean concentration	19.3	57.9	552	6970	19.9	58.5	641	7960
CV (%) ^a^	2.8	2.8	1.4	3.0	2.3	5.0	2.3	2.5
RE (%) ^b^	−3.8	−3.4	−8.1	−7.1	−0.3	−2.5	6.8	6.1
*(B) Inter-day (n = 18)*								
Mean concentration	18.9	58.2	542	6998	19.5	58.5	615	7550
CV (%)	3.1	2.6	2.3	3.0	3.9	4.2	3.6	4.6
RE (%)	−5.4	−3.1	−9.7	−6.7	−2.7	−2.4	2.5	0.7

^a^ CV (%); standard deviation/mean × 100. ^b^ (Relative error) RE (%); (calculated concentration − nominal concentration)/nominal concentration × 100. LQC: low limit of quantification. HQC: high limit of quantification.

**Table 3:** Matrix effect and recovery.

	Nominal Concentration (ng/mL)			
	3′-SL		6′-SL	
	MQC	HQC	MQC	HQC
	600	7500	600	7500
*(A) Matrix effect (IS normalized, n = 6)*				
Mean concentration (%)	113.9	103.1	101.9	108.4
CV (%) ^a^	6.4	4.5	8.8	4.2
*(B) Recovery (n = 6)*				
Mean concentration (%)	88.6	94.7	89.0	91.4
CV (%)	10.3	7.5	6.3	6.2

^a^ CV (%) = standard deviation/mean × 100.

**Table 4:** Parallelism between authentic matrix and surrogate matrix.

	Nominal Concentration (ng/mL)					
	3′-SL			6′-SL		
	LQC	MQC	HQC	LQC	MQC	HQC
	60	600	7500	60	600	7500
Endogenous concentration (ng/mL) ^a^	483	483	483	45.5	45.5	45.5
Adjusted QC concentration (ng/mL) ^b^	543	1083	7983	106	646	7546
Measured concentration (ng/mL)	501	1024	7554	97.7	651	7756
CV (%) ^c^	1.4	2.2	2.2	7.6	2.7	3.4
RE (%) ^d^	−7.8	−5.5	−5.4	−7.4	0.9	2.8

^a^ Calculated by x-intercept in the calibration curve plotted with standard addition method to the authentic matrix. ^b^ Calculated by the sum of the endogenous concentration and the nominal concentration. ^c^ CV (%); standard deviation/mean × 100. ^d^ RE (%); (calculated concentration − nominal concentration)/nominal concentration × 100. QC, quality control.

**Table 5:** Stability of biological samples.

	Nominal Concentration (ng/mL)			
	3′-SL		6′-SL	
	LQC	HQC	LQC	HQC
	481	7500	37.5	7500
*(A) Benchtop stability at room temperature (24 °C, 4 h; n = 3)*				
Mean concentration (ng/mL)	463	6890	37.0	7490
CV (%) ^a^	1.6	4.3	4.6	7.1
RE (%) ^b^	−3.7	−8.1	−1.3	−0.1
*(B)* *Post-preparative stability (10 °C, 26 h; n = 3)*				
Mean concentration (ng/mL)	482	7090	34.7	7210
CV (%)	3.3	1.6	2.8	0.6
RE (%)	0.1	−5.5	−7.4	−3.9
*(C)* *Freeze–thaw stability (4 cycles; n = 3)*				
Mean concentration (ng/mL)	465	6900	38.9	7180
CV (%)	3.2	0.5	11.5	2.2
RE (%)	−3.5	−8.0	3.9	−4.3
*(D)* *Long-term stability (−80 °C, 37 days; n = 3)*				
Mean concentration (ng/mL)	465	6750	35.4	7020
CV (%)	5.1	2.3	1.3	3.7
RE (%)	−3.4	−10.0	−5.4	−6.4

^a^ CV (%); standard deviation for the concentration/mean concentration × 100. ^b^ RE (%); (calculated concentration − nominal concentration)/nominal concentration × 100.

**Table 6:** Pharmacokinetic parameters following oral dosing of 3′-SL and 6′-SL in male rats.

	3′-SL (mg/kg)			6′-SL (mg/kg)		
	100	500	2000	100	500	2000
t_1/2_ (h)	6.5	4.9	3.4	2.8	1.8	1.8
C_max_ (ng/mL)	536 ± 105 ^a^	1100 ± 101	1930 ± 273	235 ± 2	958 ± 216	1620 ± 85
T_max_ (h)	2.0	1.0	0.5	1.0	1.0	1.0
AUC_last_ (ng·h/mL)	4000 ± 438	5700 ± 698	12000 ± 1390	991 ± 190	3270 ± 316	7620 ± 622
AUC_inf_ (ng·h/mL)	5570	7030	13,300	1050	3290	7660
CL (mL/h/kg)	18,000	71,100	150,000	95,000	152,000	261,000
V_d_ (mL/kg)	168,000	499,000	737,000	383,000	400,000	687,000

^a^ Each value expressed as mean ± standard error.

## Sample Availability

Samples of the 3′-SL and 6′-SL are not available from the authors.
